# Comparison of Three Interfacial Conductive Networks Formed in Carbon Black-Filled PA6/PBT Blends

**DOI:** 10.3390/polym13172926

**Published:** 2021-08-30

**Authors:** Hansong Li, Xinlin Tuo, Bao-Hua Guo, Jian Yu, Zhao-Xia Guo

**Affiliations:** Key Laboratory of Advanced Materials (MOE), Department of Chemical Engineering, Tsinghua University, Beijing 100084, China; lhsthu@foxmail.com (H.L.); tuoxl@mail.tsinghua.edu.cn (X.T.); bhguo@mail.tsinghua.edu.cn (B.-H.G.); yujian03@mail.tsinghua.edu.cn (J.Y.)

**Keywords:** electrical properties, interface, mechanical properties, network

## Abstract

Interfacial localization of carbon fillers in cocontinuous-structured polymer blends is well-known as a high-efficiency strategy for conductive network formation. However, a comparison with interfacial localization of carbon fillers in sea-island-structured polymer blends is lacking. Here, three types of highly efficient conductive networks formed on the basis of interfacial localization of carbon black (CB) in polyamide 6 (PA6)/poly(butylene terephthalate) (PBT) blends with different blend compositions (80/20, 50/50 and 20/80 *vol/vol*) were investigated and compared in terms of electrical resistivity, morphology as well as rheological and mechanical properties. The order of the electrical percolation threshold of CB in the three blends is 50/50 < 20/80 < 80/20, which can be attributed to different network structures. The rheological percolation thresholds are close to the electrical ones, confirming the formation of CB networks. The formation mechanisms for the three types of CB network structures are analyzed. All the three types of PA6/PBT-6 vol% CB composites showed improved tensile strength compared with PA6/PBT blends, being in favor for practical applications.

## 1. Introduction

Efficient formation of conductive filler networks in polymer matrices is key in the preparation of high-performance and low-cost conductive polymer composites [1,2,3,4,5], although other factors such as crystallization and assembly of polymers also need to be considered [6,7]. It has been known that the distribution of conductive particles, typically carbon fillers such as CB and carbon nanotubes (CNTs), in polymer matrices greatly affects the formation efficiency of conductive networks [1,4,5,8,9,10,11]. Heterogeneous particle distribution (the so-called preferential distribution) or even appropriate particle assembly is more favorable for the formation of conductive networks than homogenous particle distribution for two reasons. Firstly, only the nearest particles, not all the conductive particles, contribute to the formation of conductive networks according to the tunneling percolation model [3], and thus heterogeneous particle distribution can increase the contribution rate of conductive particles. Secondly, from the geometrical point of view, the average distance among the neighboring particles is shorter in heterogeneous particle distribution than in homogenous particle distribution [4].

The use of immiscible binary polymer blends as the matrices has been proven to be a good method for tuning the distribution of conductive particles ever since the pioneering work of Sumita et al. in 1991 [1,12,13,14,15,16,17,18,19]. It is generally recognized that the formation efficiency of the conductive networks increases to a great extent, compared with single polymer as the matrix, when carbon fillers are selectively localized in one continuous polymer phase or at the interface of a cocontinuous-structured polymer blend, according to the double percolation mechanism [12]. The interfacial localization of carbon fillers in cocontinuous-structured binary blends is considered the most ideal strategy with the highest formation efficiency of conductive networks because the interface area is small yet continuous and thus easily percolated. Although a number of successful examples have been published through kinetic control or changing the mixing thermodynamics (by grafting polymer chains on carbon fillers, for example) [19,20,21,22,23], only in a few types of blends carbon fillers can thermodynamically achieve interfacial localization [12,24,25,26,27,28]. Moreover, we noticed that in this ideal strategy, the phase continuity of the two components was considered crucial in order to ensure continuity of the interface, which is essential for achieving the second percolation [29].

Apart from cocontinuous-structure, sea-island structure is another typical phase morphology of polymer blends. However, there are considerably less investigations involving the formation of conductive networks via interfacial localization of carbon fillers in sea-island-structured polymer blends [18,24,25,28] probably because the interface is not continuous throughout the entire blend and the inter-domain distance is usually far larger than the minimum inter-particle distance (10 nm) required in the average inter-particle distance model [30]. In the previous work by our research group [26,27], we found that CB can form highly-efficient conductive networks in sea-island-structured thermoplastic polyurethane (TPU)/polyamide copolymer (COPA) and nylon-poly(m-xylene adipamide) (MXD6)/poly(ethylene terephthalate) (PET) blends through thermodynamically-driven interfacial localization. The decrease in the electrical percolation threshold can be up to 83% in the case of MXD6/PET/CB, although the average inter-domain distance deduced from the two-dimensional FESEM photos remains in the submicron level [27]. The high formation efficiency of conductive networks in MXD6/PET/CB was attributed to CB-covered PET domains serving as big conductive particles and the assistance of CB aggregates in the MXD6 phase acting as bridges. Yang et al. reported a reduction of 23% in electrical percolation threshold through interfacial distribution of CNTs in polyvinylidene difluoride (PVDF)/ethylene-octene block copolymer (OBC), and proposed that CNT-covered OBC domains assisted CNTs in the formation of the conductive networks [28].

Realizing that the interfacial strategy could be highly efficient in both cocontinuous and sea-island-structured polymer blends for the formation of conductive networks, in this work, we chose PA6/PBT/CB as a model system to investigate the effect of phase morphology on the formation of conductive networks by changing blend composition. Because the phase morphology of blends has significant effect not only on electrical conductivity, but also on other properties of the final composites (such as mechanical properties), such a study can provide a good guidance for the design of conductive composites with good overall performance by balancing various properties and cost. Scientifically, a comparison on the formation mechanisms of different types of conductive networks is of great interest. The reasons for choosing PA6/PBT/CB system are as follows: (1) The majority of CB particles are thermodynamically localized at the interface of PA6/PBT blend regardless of blend composition because CB has counterbalanced interaction with PA6 and PBT according to our preliminary research. (2) It is easy to obtain commercial PA6 and PBT resins with similar melt viscosity to avoid the effect of viscosity on phase morphology and localization of CB (Figure S1). (3) PA6 and PBT have similar melting temperature and thus similar processing window [31]. (4) PA6/PBT blends are of industrial relevance, widely used in textile industries, automotive and electronics [32]. Three blend compositions, two asymmetric (80/20 and 20/80 *vol*/*vol*) and one symmetric (50/50 *vol*/*vol*), were designed to allow a comparison between two conductive networks formed in two similar sea-island structured blends as well as a comparison between conductive networks formed in cocontinuous and sea-island-structured blends. Rheological percolation behavior of the composites was investigated to confirm the difference found in electrical percolation behavior among the three types of network structures. The three types of PA6/PBT-6 vol% CB composites showed enhanced and dramatically different dynamic modulus in the molten state and improved tensile strength compared with PA6/PBT blends.

## 2. Materials and Methods

### 2.1. Materials

Nylon 6 (PA6, 1013B), with a density of 1.14 g/cm^3^, was produced by Ube Industries, Japan. Polybutylene terephthalate (PBT, XW321a), with a density of 1.3 g/cm^3^, was produced by Sinopec Yizheng, (Yangzhou, China). Both PA6 and PBT were dried in a vacuum oven at 100 °C for 12 h before use. Carbon black (CB, VXC500), with a density of 1.84 g/cm^3^ and oil absorption value of 148 cc/100 g, was produced by Cabot Corporation (Boston, Massachusetts, USA).

### 2.2. Preparation of the Composites

A one-step melt mixing procedure was used to prepare all the composites in a torque rheometer (RH-200A, Harbin Hapro Electric Co., Ltd., Harbin, China). The compounding temperature, rotating speed and mixing time were 250 °C, 60 rpm and 5 min, respectively. The composites were denoted as PA6/PBT (x/y)-zCB, where x/y was the volume ratio of PA6 to PBT and z was the volume percentage of CB in the composite. In the expressions “zCB-filled PA6/PBT x/y” and ″PA6/PBT-zCB″, x, y and z are the same meanings.

After compounding, the composites were cut into pieces and processed into discs as test samples for electrical resistivity measurements using a hot press (LP20-B, lab Tech Engineering, Samutprakarn, Thailand). The hot pressing pressure was 50 bar, and the hot pressing temperature was 250 °C. Two types of discs were prepared: one was 0.38 mm in thickness and 75 mm in diameter for high resistivity measurements, and the other was 2.5 mm in thickness and 25 mm in diameter for other resistivity measurements.

### 2.3. Characterization

Volume resistivity measurements were carried out on the above-mentioned discs. A KDY-1 resistivity tester manufactured by Guangzhou Kunde Technology Company was used to measure samples with resistivity lower than 10^4^ Ω·cm, a ZC36 resistivity tester manufactured by Shanghai Instrument Factory was used to measure samples with volume resistivity higher than 10^10^ Ω·cm, and an ACL 800 Digital Megohmmeter (ACL, Inc., Chicago, Illinois, USA) was used to measure samples with resistivity between 10^4^ and 10^10^ Ω·cm.

For morphological investigation, the test samples were cryo-fractured in liquid nitrogen, and the cross-sectional morphology of the samples was observed with a field emission scanning electron microscope (FESEM, JEOL JSM model 7401, Tokyo, Japan) at an acceleration voltage of 5 kV. For samples that need to be etched, the PA6 phase was etched with formic acid, and the PBT phase was etched with a 15 wt% alcoholic solution of KOH. The average domain size and inter-domain distance were obtained by counting at least 6 FESEM micrographs with image analysis software of Smile View.

The distribution of CB in the blends was investigated using a transmission electron microscope (H-7650, Hitachi, Tokyo, Japan) at an acceleration voltage of 80 kV. For TEM observation, the test samples were cut into ultrathin slices (less than 100 nm in thickness) with a cryomicrotome.

Rheological measurements were carried out on a dynamic rheometer (MCR301, Anton Paar, Graz, Austria) in oscillatory shear with a sandwich fixture. Disc-shaped samples (25 mm in diameter and 1.1 mm in thickness), molded by hot-pressing at 250 °C under a pressure of 50 bar, were tested at 250 °C. The frequency is scanned from 0.01 Hz to 100 Hz with a fixed strain of 1%.

Static mechanical properties were measured using a uniaxial tensile tester (Jinjian UTM, Chengde, China) in accordance with GB/T 16421-1996 for plastics of small specimens. The dumbbell-shaped tensile bars were molded by hot-pressing at 250 °C under a pressure of 50 bar, then dried in a vacuum oven at 100 °C for 12 h and finally cooled in a desiccator containing P_2_O_5_. The stretching speed was 2 mm/min.

## 3. Results and Discussion

### 3.1. Electrical Percolation Behavior of CB in Three Different Blends

**Figure 1:**
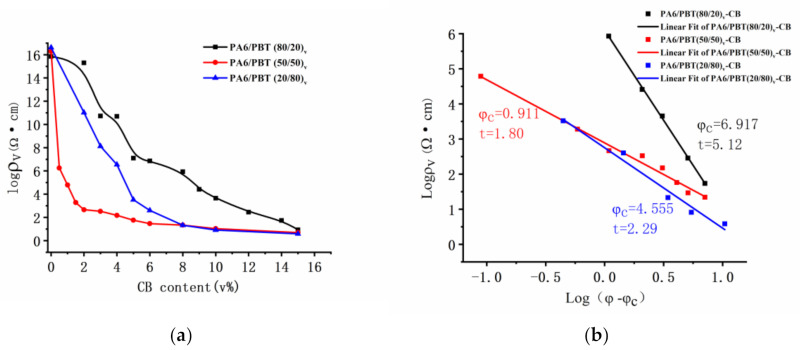
Electrical resistivity as a function of CB content (**a**), a log–log plot of electrical resistivity versus ($\varphi -\varphi_{c}$) (**b**).

The electrical percolation behavior of CB in three PA6/PBT blends with different compositions (80/20, 50/50 and 20/80 in *vol*/*vol*) is displayed in Figure 1a, showing the formation of conductive networks at different CB loadings. As shown in Figure 1b, the electrical percolation thresholds deduced from the curves fitting to the power law (i.e.,$\;\sigma =\sigma_{0}{\left(\varphi -\varphi_{c}\right)}^{t}$) are 6.92, 0.91 and 4.56 vol% for PA6/PBT blend compositions of 80/20, 50/50 and 20/80, respectively. They are all dramatically lower than that of CB in neat PA6 (>15 vol%, Figure S2), and that in PA6/PBT 20/80 blend is lower than that in neat PBT (5.96 vol%). The electrical percolation threshold of CB in the symmetric blend (50/50) is the lowest (only 0.91 vol%), emphasizing the importance of the small interface area and interface continuity in the formation of conductive networks. Although in the two asymmetric blends (80/20 and 20/80), the blend composition is only inverse, the electrical percolation threshold of CB in 20/80 blend is significantly lower than in 80/20 blend. This is rather surprising but can be reasonably understood by the difference in the CB network structures after morphological investigation.

The critical exponent t values are 5.12, 1.80 and 2.29 for composites having PA6/PBT blend compositions of 80/20, 50/50 and 20/80, respectively. This is understandable. According to the classical percolation theory, t value depends on the connectivity of the system [3] and universal values of 1.3 and 2 were predicted for two and three-dimensional networks [1,3]. In reality, a wide range of t values (1–12) have been reported [1]. A comparison of the three t values in this work reveals that t is the smallest for CB-filled PA6/PBT 50/50 blend because of homogeneous distribution of CB aggregates at the continuous PA6/PBT interface, and a much higher t value found for CB-filled 80/20 blend suggests a much broader tunneling distance distribution [1] caused by the formation of co-supporting networks with two types of conductive fillers: CB-covered PBT domains and CB aggregates in the matrix phase.

### 3.2. Comparison of the Composite Morphologies

The CB network structure is directly related to phase morphology of PA6/PBT blends and localization of CB. The phase morphology of the three types of PA6/PBT blends (50/50, 80/20 and 20/80) without CB were verified by FESEM after itching PBT or PA6 phase with appropriate solvent. As shown in Figure S3, the 50/50 blend is cocontinuous whereas 80/20 and 20/80 blends exhibit a typical sea-island structure, as predicted from the blend composition.

**Figure 2:**
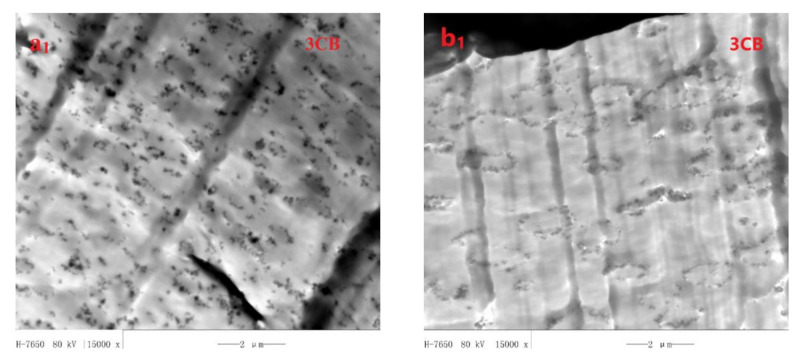

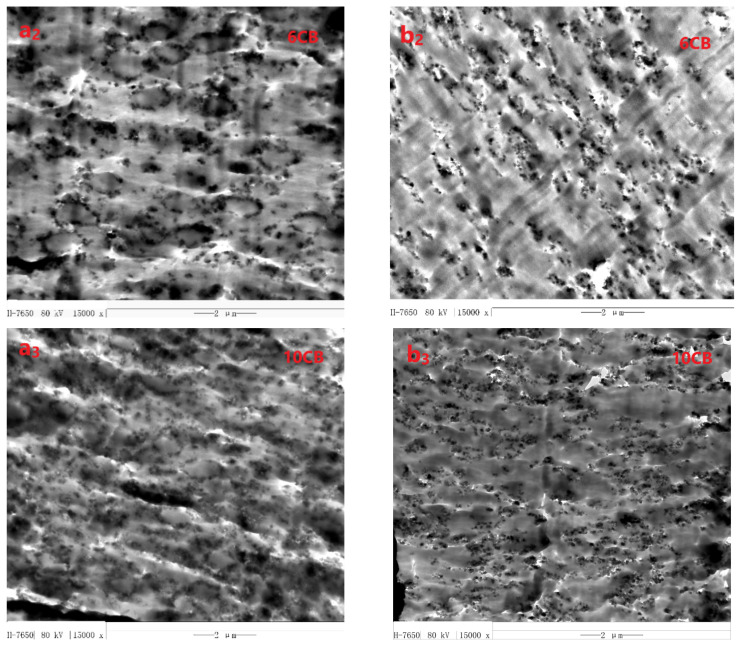
TEM micrographs of PA6/PBT(80/20)-CB (**a_1_**,**a_2_**,**a_3_**) and PA6/PBT(20/80)-CB (**b_1_**,**b_2_**,**b_3_**) composites containing different CB contents.

**Figure 3:**
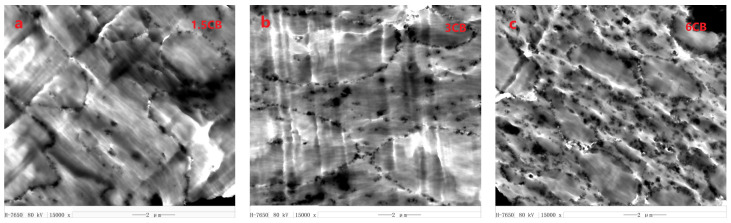
TEM micrographs of PA6/PBT(50/50)-CB composites containing different CB contents.

The localization of CB in the three types of PA6/PBT blends was investigated by TEM, and the micrographs are presented in Figure 2 and Figure 3. It is clear that, at low CB loadings, the majority of CB particles are selectively localized at PA6/PBT interface. At high CB loadings, excess CB particles are observed in the PA6 phase, regardless of blend composition. Even at high CB loadings, CB particles are hardly observed in the PBT phase no matter if it is the matrix or dispersed phase. These observations reveal that CB has almost counterbalanced interaction with PA6 and PBT, with very slightly higher affinity to PA6. This was also confirmed by FESEM micrographs of 3CB-filled PA6/PBT 80/20 and 20/80 blends (Figure S4), where CB particles were observed on the PA6 side of the dispersed phase, i.e., at the bottoms of the holes in 80/20 blend and on the surfaces of PA6 spheres in 20/80 blend. It is not surprising that, at high CB loadings, the interface is CB-saturated, and excess CB particles are found in the phase with which CB has slightly higher affinity (in this case, PA6 phase). A similar phenomenon was also observed in CB-filled TPU/COPA, where excess CB aggregates were found in the COPA phase at high CB loadings [26]. In combination with our previous work [8,26,27], it is believed that absolute counterbalanced interaction does not exist, and among the commonly known polymers, nylons have the highest interaction level with CB, although CB particles, at low loadings, can achieve interfacial localization in nylon/TPU or nylon/aromatic polyester (PET or PBT) blends. The preference of CB for nylons may be related to the abundant amide bonds in the molecular chains of nylons. Because there are numerous polar groups (such as hydroxyl, carboxyl and carbonyl groups) on the surface of CB [27], nylons can form hydrogen bonds with CB via both C=O and N-H sites of amide bonds, and thus strong hydrogen bonding is formed between nylon and CB. This was confirmed by a shift of both amide I and II bands in the FTIR spectra of MXD6/PET/CB [27] and PA6/PBT/CB composite (Figure S5). Aromatic polyesters (PET and PBT) have slightly weaker interaction with CB than nylons (MXD6 and PA6) because they can form hydrogen bonds with CB via ester linkage and π-π interaction with CB via aromatic rings.

It is noted that, for CB-filled 50/50 blend, the interface is almost fully covered by CB particles with as low as 1.5 vol% of CB, being in agreement with the ultralow electrical percolation of CB; whereas, for CB-filled 80/20 blend, the interface is far from complete coverage by CB even with 3 vol% CB, and the coverage rate of the interface increases with increasing CB loading. For CB-filled 20/80 blend, a strong impression is the heterogeneous distribution of the CB-covered PA6 domains acting as big conductive particles, being in favor for the formation of conductive networks by shortening inter-particle distance. This can be interpreted by the dramatic increase in the viscosity of the PA6 phase with a large amount (much larger in term of CB concentration than in 80/20 blend) of excess CB particles inside, which inhibits the mobility of the dispersed PA6 phases.

**Figure 4:**
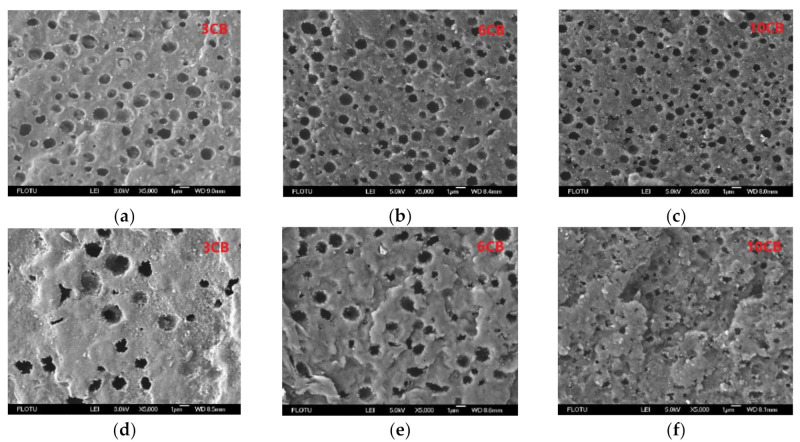
FESEM photos of PA6/PBT(80/20)-CB composites with different CB contents: (**a**) 3 vol%, (**b**) 6 vol%, (**c**) 10 vol%; and PA6/PBT(20/80)-CB composites with different CB contents: (**d**) 3 vol%, (**e**) 6 vol% and (**f**) 10 vol%. The PBT phase in PA6/PBT(80/20)-CB composites was etched by alcoholic solution of KOH, and the PA6 phase in PA6/PBT(20/80)-CB composites was etched by formic acid.

**Figure 5:**
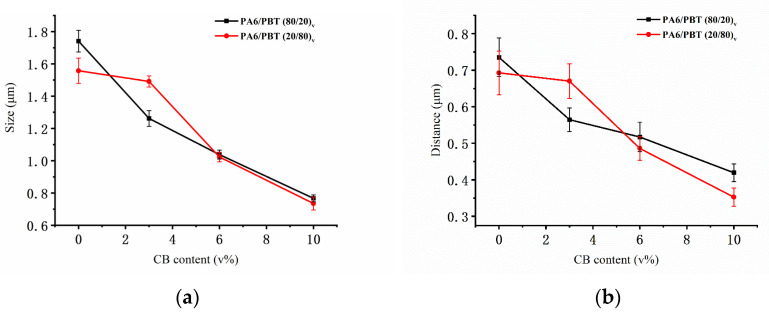
Average domain size (**a**) and average inter-domain distance (**b**) as a function of CB content in PA6/PBT(80/20)-CB and PA6/PBT(20/80)-CB composites.

In CB-filled asymmetric blends (80/20 and 20/80), the domain size and inter-domain distance are two crucial parameters affecting the formation of conductive networks because the CB-covered domains act as micron-sized conductive particles, much bigger than CB aggregates. Thus, they were measured by carrying out FESEM observation after itching domains with appropriate solvent. The FESEM micrographs are shown in Figure 4, and the quantitative data are presented in Figure 5. For both 80/20 and 20/80 series, both domain size and inter-domain distance decrease with increasing CB loading, implying that CB has compatibilization effect on the blends because of its interfacial localization. At high CB loadings (6 and 10 vol%), the domain size is similar for both series, but the inter-domain distance is smaller for 20/80 blend, being in agreement with the heterogeneous distribution of PA6 domains observed by TEM. According to the values of electrical percolation thresholds of CB in the two types of blends (6.92 vol% and 4.56 vol%), at 6 vol% CB, the conductive networks are about to form in 80/20 blend, and they have already formed in 20/80 blend. The average domain size is around 1 μm, and the average inter-domain distance is around 0.5 μm for both types of blends. The latter is at a similar level to those found in our previous work on MXD6/PET/CB and TPU/COPA/CB composites [26,27], i.e., it is much larger than 10 nm, the inter-particle distance used in the average inter-particle distance model [30].

### 3.3. Rheological Percolation Behavior of CB in Three Different Blends

Rheology is an effective mean to investigate the construction of filler networks in a polymer matrix because the rheological properties of the composite melt change dramatically upon percolation of the filler [33,34,35,36]. Therefore, frequency-weep testing was carried out on all of the three series of composites with different CB contents, and the results are given in Figures S6–S8. Clearly, for almost all of the samples, all three rheological parameters, i.e., the storage modulus (G’), loss modulus (G”) and complex viscosity (|η*|), show frequency-dependence over the whole frequency range. G’ and G” increase when the frequency increases; whereas |η*| shows obvious shear thinning behavior.

**Figure 6:**
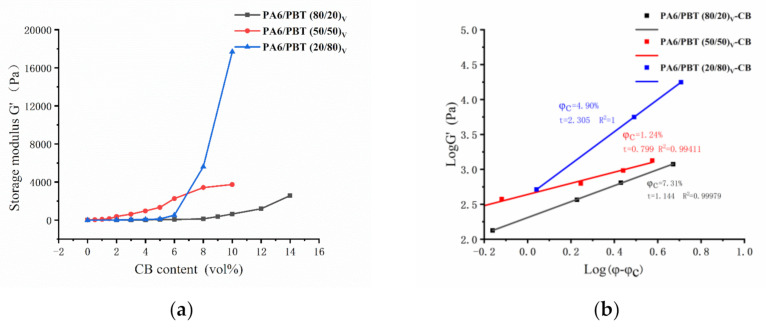
Storage modulus G’as a function of CB content (**a**), a log–log plot of storage modulus versus ($\varphi -\varphi_{c}$) (**b**).

It has been reported that the power law relation predicted by the statistical percolation theory holds for rheological data [33,37]. G’ values at low frequencies are often used to deduce the rheological percolation values of fillers because elastic load transfer is more sensitive to the formation of filler networks than viscous dissipation, although G” and |η*| values at low frequencies could also fit to the scaling law of the percolation theory [33,37]. Thus, the G’ values at 0.01 Hz as a function of CB content are plotted in Figure 6a, and the curves fitting to the power law are shown in Figure 6b. Because of the reinforcement effect of rigid CB particles, G’ increases slightly at low CB loadings for all three types of composites. A huge increase occurs when CB percolates at a higher CB loading. It is noted that the adjusted correlation coefficients (R^2^) in Figure 6b are all high enough to guarantee the linear relationship of log G’-log(φ-φ_c_). The rheological percolation thresholds of CB in PA6/PBT 80/20, 50/50 and 20/80 blends are 7.31, 1.24 and 4.90 vol%, respectively, being close to the values of the electrical percolation thresholds. In the literature, some authors reported lower rheological percolation thresholds than electrical percolation thresholds [34,37,38] and attributed the difference to different percolation mechanism that require different inter-particle distance (i.e., different network density), although both rheological and electrical networks are formed through a non-contact mode [37]. In most cases, the electrical resistivities were measured in the solid state, whereas the rheological measurements were carried out in the molten state, and thus the comparison may not be strict [37]. Santamaria’s group investigated the electrical and rheological percolations of MWCNTs and graphene in molten thermoplastic polyurethane and found that the electrical percolation threshold of MWCNTs at a frequency of 20 Hz was lower than rheological percolation threshold whereas practically the same values were obtained in the case of graphene [39,40]. Therefore, it seems difficult to draw a general trend.

It is noted that the G’ values at 8 and 10 vol% CB for 50/50 blend-based composites are much smaller than those for 20/80 blend-based composites, although the CB percolates at very low content (1.24 vol%) in 50/50 blend, indicating that the CB networks are much less elastic in 50/50 blend than in 20/80 blend at high level of CB contents. This phenomenon is related to their different network structures and different contribution level of the excess CB, which will be discussed in the following section.

### 3.4. Comparison of the Formation Mechanisms of Different Network Structures

**Figure 7:**
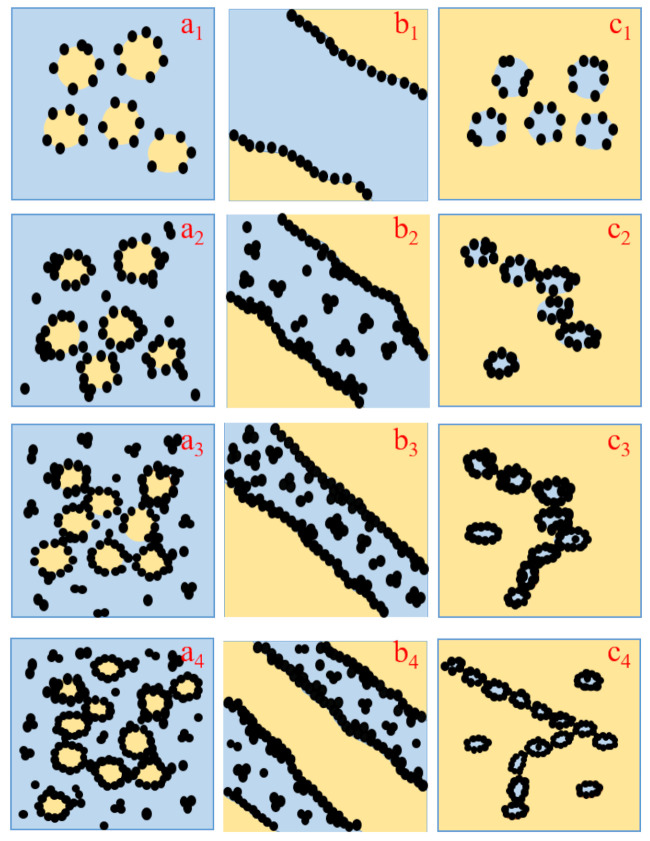
Illustration of the proposed mechanisms for the formation of different percolated CB networks in three different PA6/PBT blends: 80/20 (**a_1_**,**a_2_**,**a_3_**,**a_4_**), 50/50 (**b_1_**,**b_2_**,**b_3_**,**b_4_**) and 20/80 (**c_1_**,**c_2_**,**c_3_**,**c_4_**).

As discussed above, the electrical and rheological investigations consistently show formation of percolated CB networks at different CB levels in the three different PA6/PBT blends. This can be attributed to the formation of different network structures. On the basis of morphological observation as well as electrical and rheological data, the mechanisms for the formation of different percolated CB networks are proposed and illustrated in Figure 7.

At a low CB loading of 1 vol% (Figure 7(a_1_,b_1_,c_1_)), almost all the CB particles are selectively localized at PA6/PBT interface in all the three types of blends regardless of blend morphology, because they adsorb both types (PA6 and PBT) of molecules during melt compounding and behave similar to Janus particles as a result of strong interaction with both PA6 and PBT molecules via hydrogen bonding and π-π stacking [27,41]. However, the difference among the three cases is obvious. The interface of 50/50 blend is fully covered with CB particles, whereas those of 80/20 and 20/80 blends are only partly covered and far from full coverage. This was caused by the size effect of the second phase. The interface area of a co-continuous blend is much smaller than the total interface area of dispersed domains. Thus, in 1 CB-filled 50/50 blend, CB forms a percolated network, whereas in the other two systems, there is not any network formation. As a consequence, the electrical resistivity of PA6/PBT(50/50)-1CB composite drops down to 10^5^ Ω·cm, whereas those of PA6/PBT(80/20)-1CB and PA6/PBT(20/80)-1CB remain very high (around 10^15^ Ω·cm).

At 5 vol% CB (Figure 7(a_2_,b_2_,c_2_)), in the cases of 80/20 and 20/80 blends, the majority of CB particles are localized on the surface of the dispersed phase and percolation of CB in the surface area of the domains (i.e., the primary percolation) is achieved. The CB-covered domains must be highly conductive and play the role of micron-sized conductive fillers. Percolated CB networks are formed in 20/80 blend but not in 80/20 blend because the inter-domain distance is smaller in 20/80 blend than in 80/20 blend and can allow electron transfer through tunneling, and thus double percolation occurs. As to 50/50 blend, a significant number of CB particles are localized in the PA6 phase because the small interface area cannot allow localization of such “large amount” of CB particles and also because PA6 has slightly higher affinity to CB than PBT, as discussed above. The electrical resistivity order is: PA6/PBT(50/50)-5CB < PA6/PBT(20/80)-5CB < PA6/PBT(80/20)-5CB, and the G’ order is inverse, revealing that the electrical and rheological data are in accordance.

When CB loading increases to 7 vol% (Figure 7(a_3_,b_3_,c_3_)), in the case of 80/20 blend, a significant number of CB particles are localized in the PA6 phase because the interface is fully impregnated by CB, and those between two neighboring domains can play a role of “electrical bridges” for the micron-sized conductive particles (CB-covered domains). Thus, the secondary percolation occurs, involving two types of conductive particles: CB-covered PBT domains as micron-sized conducive particles and CB aggregates in the matrix phase. PA6/PBT(80/20)-7CB composite is percolated through the two-level percolation mechanism [27,42]. As to 20/80 blend, the excess CB particles are localized in the PA6 domains; most of them are observed alongside the interface, increasing the thickness of the interface.

At a high CB loading of 10 vol% (Figure 7(a_4_,b_4_,c_4_)), the electrical resistivity of PA6/PBT(50/50)-10CB and PA6/PBT(20/80)-10CB is similar, whereas G’ of PA6/PBT(20/80)-10CB is greater than that of PA6/PBT(50/50)-10CB, although CB in the 50/50 blend has a much lower percolation threshold than in 20/80 blend. This can be attributed to the different localization and thus different level of contribution of excess CB particles. In the 50/50 blend, a significant number of additional CB particles localize in the nylon phase, and they do not contribute to the formation of network structure; whereas the additional CB particles alongside the PA6/PBT(20/80) interface has a strong contribution to the network structure. Therefore, the CB network in PA6/PBT(20/80)-10CB melt is more elastic than in PA6/PBT(50/50)-10CB melt, although the CB networks are well established in both composites. Similar electrical and different G’ values are understandable because electrical and rheological behaviors involve different mechanisms: electron hopping through tunneling and stress transfer through polymer chains as bridges, respectively. Both the electrical conductivity and G’ values of PA6/PBT(80/20)-10CB are considerably lower than those of PA6/PBT(20/80)-10CB and PA6/PBT(50/50)-10CB, suggesting that the CB networks in 80/20 blend are much less perfect than those in 20/80 and 50/50 blends because 10 vol% CB is just above the percolation threshold (around 7 vol%) of 80/20 system, whereas it is well beyond the percolation thresholds of 20/80 and 50/50 systems (around 5 and 1 vol%).

### 3.5. Mechanical Properties of the Composites

**Figure 8:**
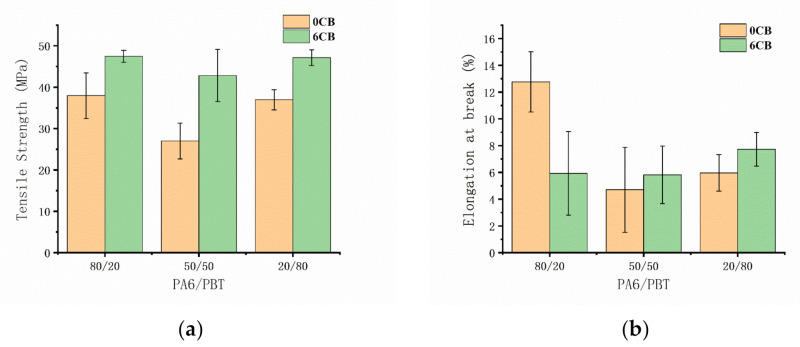
Tensile strengths (**a**) and elongations at break (**b**) of PA6/PBT-CB composites with CB contents of 0 and 6 vol%.

The static mechanical properties of the three types of composites with a constant CB content of 6 vol% were investigated by uniaxial tensile testing and compared with those of the corresponding PA6/PBT blends. As shown in Figure 8 and Figure S9, blending of PA6 and PBT leads to significant decrease in both tensile strength and elongation at break because of the immiscibility of the two components. Upon incorporation of 6 vol% CB, the tensile strength of all of the blends increases, whereas all of the blends still exhibit brittle behavior. This reveals that CB has a strengthening effect on the blends for all three compositions, although CB network structure is different in the three blends.

Being a commercially available cheap filler, CB is widely used as a conductive filler to improve electrical or thermal conductivity or antistatic property of non-conductive polymers, or as a reinforcing filler to improve dimensional stability and wear characteristics, or as a colorant, antioxidant and light stabilizer [43]. However, the incorporation of CB to a polymer (or polymer blend) may not necessarily increase tensile strength, depending on various factors such as the type of the polymer, polymer/CB interaction and CB loading [43,44,45]. The increase in tensile strength of PA6/PBT/CB composites discovered in this work may be attributed to good and almost counterbalanced PA6-CB and PBT-CB interactions as well as the interfacial CB networks. A potential application area of the strategies investigated in this work is recycling. It means that PA6 and PBT can be recycled into conductive or antistatic materials in any proportion by melt-mixing with a small amount of CB.

## 4. Conclusions

CB particles can form different interfacial network structures in PA6/PBT blends, depending on the blend composition. All the three typical phase morphologies investigated in this work, i.e., sea-island, cocontinuous and inverse sea-island structures, corresponding to blend compositions of 80/20, 50/50 and 20/80, can allow formation of high-efficiency interfacial CB networks, as revealed by the much lower electrical percolation thresholds of CB compared to that in neat PA6. In the symmetric 50/50 blend with cocontinuous phase morphology, the electrical percolation threshold of CB is the lowest (0.94 vol%) because the PA6/PBT interface area is small yet continuous. An interesting phenomenon is that CB has a much lower electrical percolation threshold in asymmetric 20/80 blend than in 80/20 blend (4.56 vs. 6.92 vol%), which can be attributed to the shorter inter-domain distance caused by inhomogeneous distribution of PA6 domains. The rheological percolation thresholds of CB in PA6/PBT 80/20, 50/50 and 20/80 blend melts are close to the corresponding electrical percolation thresholds, confirming the formation of CB networks. The difference among the three CB network structures can be reflected not only by their different electrical and rheological percolation threshold values but also by their difference in electrical or rheological data at high CB loadings. All of the three types of interfacial CB network structures show strengthening effect on the blends.

## Data Availability

Data are contained within the article or Supplementary Material.

## Supplementary Materials

The following are available online at https://www.mdpi.com/article/10.3390/polym13172926/s1, Figure S1: Complex viscosity of virgin PA6 and PBT resins at 250 °C as a function of frequency; Figure S2: Electrical resistivity as a function of CB content for PA6/CB and PBT/CB composites. Figure S3: FESEM micrographs of different PA6/PBT blends: 80/20 (a), 50/50 (b) and 20/80 (c). In a and b, PBT was etched with alcoholic solution of KOH; while in c, PA6 domains were etched with formic acid. Figure S4: FESEM photos of PA6/PBT(80/20)-3CB (a) and PA6/PBT(20/80)-3CB (b) composites, showing selective localization of CB particles at the interface. Figure S5: FTIR spectra of PA6/PBT(80/20)-0CB and PA6/PBT(80/20)-10CB composites. The amide I and II bands shifted from 1638.1 to 1635.1 cm^−1^ and from 1543.4 to 1539.1 cm^−1^, and the carbonyl peak shifted from 1713.4 to 1710.1 cm^−1^ after adding 10 vol% CB. Figure S6: Storage modulus G’ as a function of frequency for PA6/PBT(80/20)-CB (a), PA6/PBT(50/50)-CB (b), and PA6/PBT(20/80)-CB (c) composites with different CB contents. Figure S7: Loss modulus G″ as a function of frequency for PA6/PBT(80/20)-CB (a), PA6/PBT(50/50)-CB (b), and PA6/PBT(20/80)-CB (c) composites with different CB contents. Figure S8: Complex viscosity |η*| as a function of frequency for PA6/PBT(80/20)-CB (a), PA6/PBT(50/50)-CB (b), and PA6/PBT(20/80)-CB (c) composites with different CB contents. Figure S9: Tensile strengths (a) and elongations at break (b) of PA6 and PBT with CB contents of 0 and 6 vol%.

## References

[1] Deng H., Lin L., Ji M., Zhang S., Yang M., Fu Q. (2014). Progress on the morphological control of conductive network in conductive polymer composites and the use as electroactive multifunctional materials. Prog. Polym. Sci..

[2] Pang H., Xu L., Yan D.-X., Li Z.-M. (2014). Conductive polymer composites with segregated structures. Prog. Polym. Sci..

[3] Mutiso R.M., Winey K.I. (2015). Electrical properties of polymer nanocomposites containing rod-like nanofillers. Prog. Polym. Sci..

[4] Gulrez S.K.H., Ali Mohsin M.E., Shaikh H., Anis A., Pulose A.M., Yadav M.K., Qua E.H.P., Al-Zahrani S.M. (2014). A review on electrically conductive polypropylene and polyethylene. Polym. Compos..

[5] Zhang M., Li Y., Su Z., Wei G. (2015). Recent advances in the synthesis and applications of graphene–polymer nanocomposites. Polym. Chem..

[6] Yuan T., Sun Z., Mu A.U., Zeng M., Kalin A.J., Cheng Z., Olson M.A., Fang L. (2018). Assembly and Chiral Memory Effects of Dynamic Macroscopic Supramolecular Helices. Chem. Eur. J..

[7] Zhu L., Wang H., Liu M., Jin Z., Zhao K. (2018). Effect of Core-Shell Morphology on the Mechanical Properties and Crystallization Behavior of HDPE/HDPE-g-MA/PA6 Ternary Blends. Polymers.

[8] Zhang Q., Wang J., Zhang B.-Y., Guo B.-H., Yu J., Guo Z.-X. (2019). Improved electrical conductivity of polymer/carbon black composites by simultaneous dispersion and interaction-induced network assembly. Compos. Sci. Technol..

[9] Zhang Q., Zhang B.-Y., Guo B.-H., Guo Z.-X., Yu J. (2020). High-temperature polymer conductors with self-assembled conductive pathways. Compos. Part B Eng..

[10] Maiti S., Bera R., Karan S.K., Paria S., De A., Khatua B.B. (2019). PVC bead assisted selective dispersion of MWCNT for designing efficient electromagnetic interference shielding PVC/MWCNT nanocomposite with very low percolation threshold. Compos. Part B Eng..

[11] Li Y., Huang X., Zeng L., Li R., Tian H., Fu X., Wang Y., Zhong W.-H. (2019). A review of the electrical and mechanical properties of carbon nanofiller-reinforced polymer composites. J. Mater. Sci..

[12] Sumita M., Sakata K., Asai S., Keizo M. (1991). Dispersion of fillers and the electrical conductivity of polymer blends filled with carbon black. Polym. Bull..

[13] Duan L., Fu S., Deng H., Zhang Q., Wang K., Chen F., Fu Q. (2014). The resistivity–strain behavior of conductive polymer composites: Stability and sensitivity. J. Mater. Chem. A.

[14] Zhang S., Deng H., Zhang Q., Fu Q. (2014). Formation of Conductive Networks with Both Segregated and Double-Percolated Characteristic in Conductive Polymer Composites with Balanced Properties. ACS Appl. Mater. Interfaces.

[15] Bilotti E., Zhang R., Deng H., Baxendale M., Peijs T. (2010). Fabrication and property prediction of conductive and strain sensing TPU/CNT nanocomposite fibres. J. Mater. Chem..

[16] Bilotti E., Zhang H., Deng H., Zhang R., Fu Q., Peijs T. (2013). Controlling the dynamic percolation of carbon nanotube based conductive polymer composites by addition of secondary nanofillers: The effect on electrical conductivity and tuneable sensing behaviour. Compos. Sci. Technol..

[17] Zonder L., Ophir A., Kenig S., McCarthy S. (2011). The effect of carbon nanotubes on the rheology and electrical resistivity of polyamide 12/high density polyethylene blends. Polymer.

[18] Wang X., Xu L., Xu X.-B., Chen S., Wang Y.-J. (2011). Effect of Mixing Process and Morphologies on the Electrical Conductivity of PA6/EVA/CB Composites. Polym. Plast. Technol. Eng..

[19] Zhai W., Zhao S., Wang Y., Zheng G., Dai K., Liu C., Shen C. (2018). Segregated conductive polymer composite with synergistically electrical and mechanical properties. Compos. Part A Appl. Sci. Manuf..

[20] Chen J., Cui X., Zhu Y., Jiang W., Sui K. (2017). Design of superior conductive polymer composite with precisely controlling carbon nanotubes at the interface of a co-continuous polymer blend via a balance of π-π interactions and dipole-dipole interactions. Carbon.

[21] Shen L., Wang F., Jia W., Yang H. (2012). Thermodynamically induced self-assembled electrically conductive networks in carbon-black-filled ternary polymer blends. Polym. Int..

[22] Al-Saleh M.H., Sundararaj U. (2008). An innovative method to reduce percolation threshold of carbon black filled immiscible polymer blends. Compos. Part A Appl. Sci. Manuf..

[23] Tan Y., Fang L., Xiao J., Song Y., Zheng Q. (2013). Grafting of copolymers onto graphene by miniemulsion polymerization for conductive polymer composites: Improved electrical conductivity and compatibility induced by interfacial distribution of graphene. Polym. Chem..

[24] Baudouin A.-C., Devaux J., Bailly C. (2010). Localization of carbon nanotubes at the interface in blends of polyamide and ethylene–acrylate copolymer. Polymer.

[25] Baudouin A.-C., Auhl D., Tao F., Devaux J., Bailly C. (2011). Polymer blend emulsion stabilization using carbon nanotubes interfacial confinement. Polymer.

[26] Zhang Q., Wang J., Yu J., Guo Z.-X. (2017). Improved electrical conductivity of TPU/carbon black composites by addition of COPA and selective localization of carbon black at the interface of sea-island structured polymer blends. Soft Matter.

[27] Li H., Zhang Q., Guo B.-H., Guo Z.-X., Yu J. (2019). Conductive nylon-MXD6 composites prepared by melt compounding associated with formation of carbon black-covered PET domains serving as big conductive particles. Polymer.

[28] Zha X.-J., Pu J.-H., Ma L.-F., Li T., Bao R.-Y., Bai L., Liu Z.-Y., Yang M.-B., Yang W. (2018). A particular interfacial strategy in PVDF/OBC/MWCNT nanocomposites for high dielectric performance and electromagnetic interference shielding. Compos. Part A Appl. Sci. Manuf..

[29] Zhang Z., Cao M., Chen P., Yang B., Wu B., Miao J., Xia R., Qian J. (2019). Improvement of the thermal/electrical conductivity of PA6/PVDF blends via selective MWCNTs-NH2 distribution at the interface. Mater. Des..

[30] Li J., Ma P.C., Chow W.S., To C.K., Tang B.Z., Kim J.-K. (2007). Correlations between Percolation Threshold, Dispersion State, and Aspect Ratio of Carbon Nanotubes. Adv. Funct. Mater..

[31] Jubinville D., Chang B.P., Pin J.-M., Mohanty A.K., Misra M. (2019). Synergistic thermo-oxidative maleation of PA11 as compatibilization strategy for PA6 and PBT blend. Polymer.

[32] Scaffaro R., Botta L., La Mantia F.P., Magagnini P., Acierno D., Gleria M., Bertani R. (2005). Effect of adding new phosphazene compounds to poly(butylene terephthalate)/polyamide blends. I: Preliminary study in a batch mixer. Polym. Degrad. Stab..

[33] Arboleda-Clemente L., Ares-Pernas A., García X., Dopico S., Abad M.J. (2017). Segregated conductive network of MWCNT in PA12/PA6 composites: Electrical and rheological behavior. Polym. Compos..

[34] Bitenieks J., Merijs Meri R., Zicans J., Buks K. (2020). Dynamic Mechanical, Dielectrical, and Rheological Analysis of Polyethylene Terephthalate/Carbon Nanotube Nanocomposites Prepared by Melt Processing. Int. J. Polym. Sci..

[35] Jiang C., Han S., Chen S., Zhou H., Wang X. (2021). The role of PTFE in-situ fibrillation on PET microcellular foaming. Polymer.

[36] Hadaeghnia M., Ahmadi S., Ghasemi I., Wood-Adams P.M. (2020). Manipulating the morphology of PA6/POE blends using graphene to achieve balanced electrical and mechanical properties. Compos. Sci. Technol..

[37] Gao C., Liu P., Ding Y., Li T., Wang F., Chen J., Zhang S., Li Z., Yang M. (2018). Non-contact percolation of unstable graphene networks in poly(styrene-co-acrylonitrile) nanocomposites: Electrical and rheological properties. Compos. Sci. Technol..

[38] Du F., Scogna R.C., Zhou W., Brand S., Fischer J.E., Winey K.I. (2004). Nanotube Networks in Polymer Nanocomposites: Rheology and Electrical Conductivity. Macromolecules.

[39] Fernández M., Landa M., Muñoz M.E., Santamaria A. (2011). Electrical conductivity of PUR/MWCNT nanocomposites in the molten state, during crystallization and in the solid state. Eur. Polym. J..

[40] Canales J., Muñoz M.E., Fernández M., Santamaría A. (2016). Rheology, electrical conductivity and crystallinity of a polyurethane/graphene composite: Implications for its use as a hot-melt adhesive. Compos. Part A Appl. Sci. Manuf..

[41] Omurtag P.S., Alkan B., Durmaz H., Hizal G., Tunca U. (2018). Indirect functionalization of multiwalled carbon nano tubes through non-covalent interaction of functional polyesters. Polymer.

[42] Hoseini A.H.A., Arjmand M., Sundararaj U., Trifkovic M. (2017). Tunable electrical conductivity of polystyrene/polyamide-6/carbon nanotube blend nanocomposites via control of morphology and nanofiller localization. Eur. Polym. J..

[43] Huang J.-C. (2002). Carbon black filled conducting polymers and polymer blends. Adv. Polym. Technol..

[44] Hu J., Zhang H.-B., Hong S., Jiang Z.-G., Gui C., Li X., Yu Z.-Z. (2014). Simultaneous Improvement in Both Electrical Conductivity and Toughness of Polyamide 6 Nanocomposites Filled with Elastomer and Carbon Black Particles. Ind. Eng. Chem. Res..

[45] Mondal S., Ganguly S., Das P., Khastgir D., Das N.C. (2017). Low percolation threshold and electromagnetic shielding effectiveness of nano-structured carbon based ethylene methyl acrylate nanocomposites. Compos. Part. B Eng..

