# Supplementary material for: Comparison of Three Interfacial Conductive Networks Formed in Carbon Black-Filled PA6/PBT Blends
Source: Polymers (Basel). 2021 Aug 30;13(17):2926. doi: 10.3390/polym13172926 (PMC8434115; doi:10.3390/polym13172926)
Supplement: Supplementary file 1 [file polymers-13-02926-s001.zip › polymers-1354448-SI.pdf]

Supplemental files

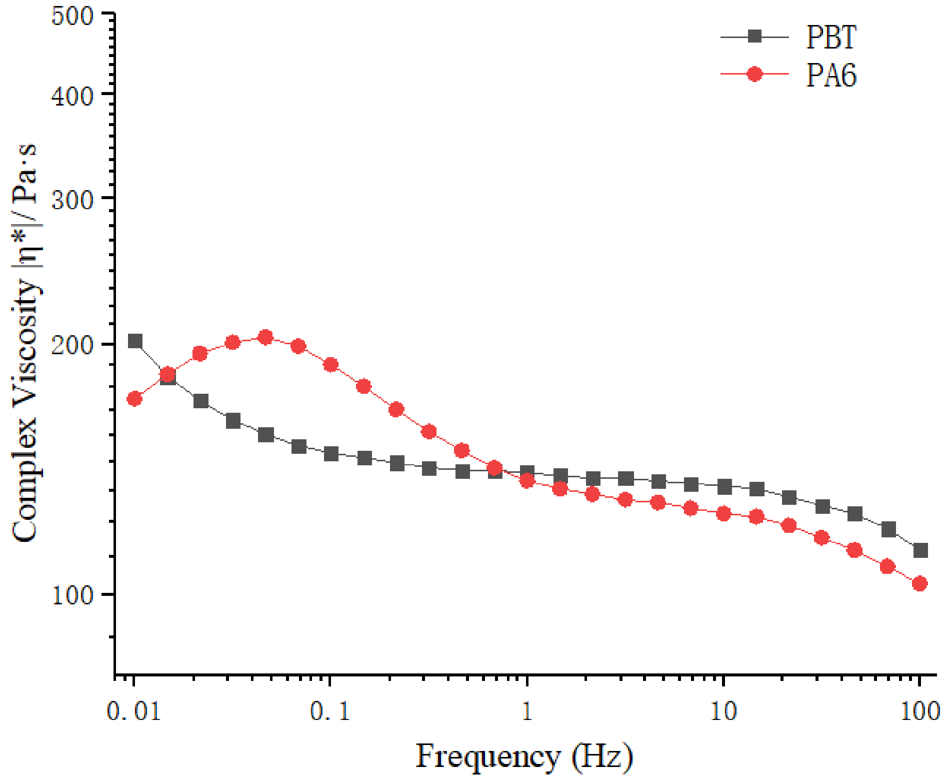

Figure S1. Complex viscosity of virgin PA6 and PBT resins at 250 ℃ as a function of frequency.

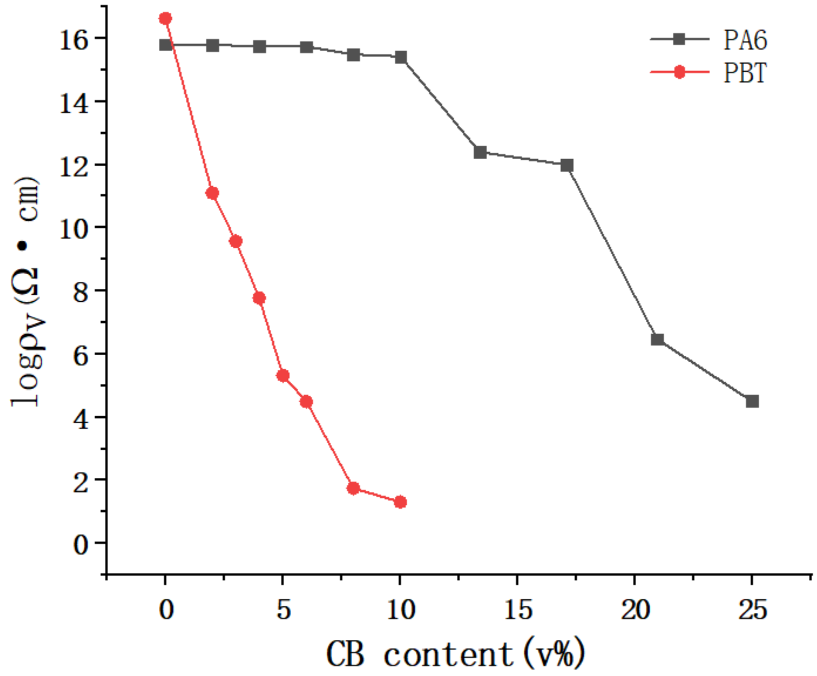

Figure S2. Electrical resistivity as a function of CB content for PA6/CB and PBT/CB composites.

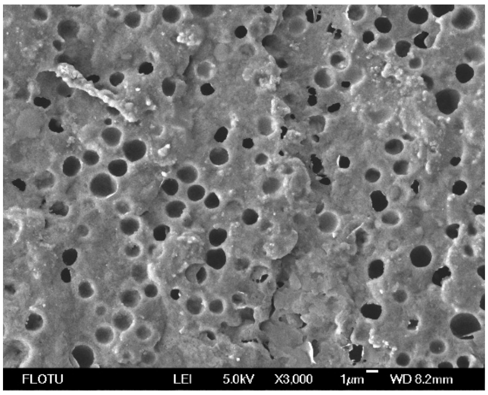

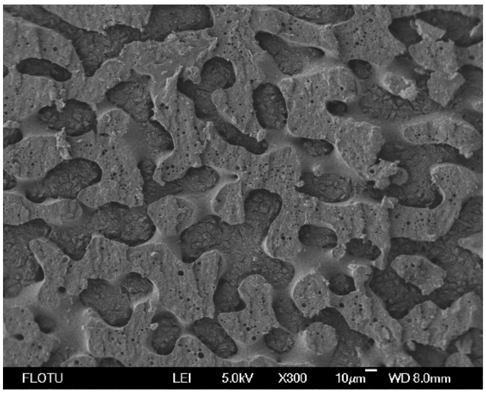

(a) (b)

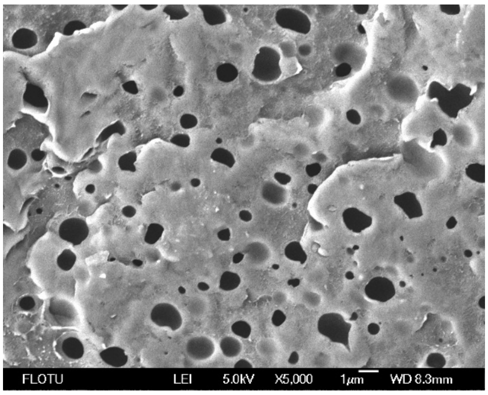

(c)

Figure S3. FESEM micrographs of different PA6/PBT blends: 80/20 (a), 50/50 (b) and 20/80 (c). In a and b, PBT was etched with alcoholic solution of KOH; while in c, PA6 domains were etched with formic acid.

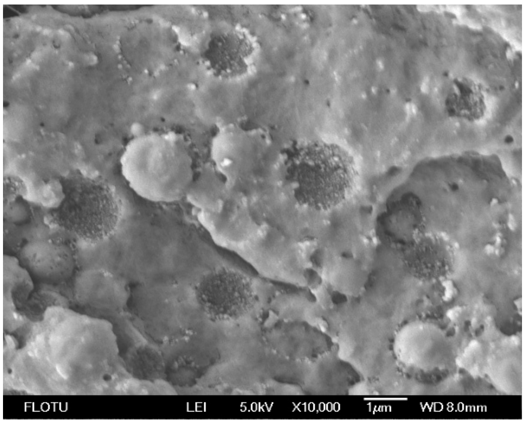

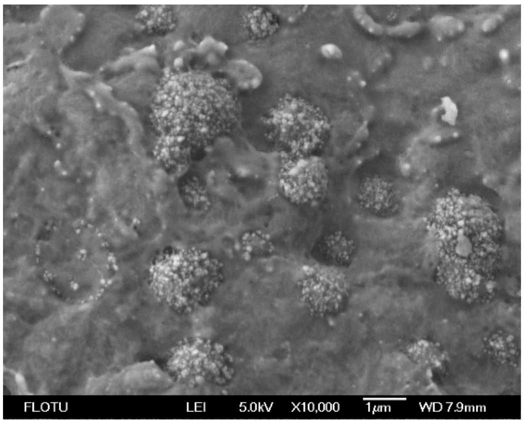

Figure S4. FESEM photos of PA6/PBT(80/20)-3CB (a) and PA6/PBT(20/80)-3CB (b) composites, showing selective localization of CB particles at the interface.

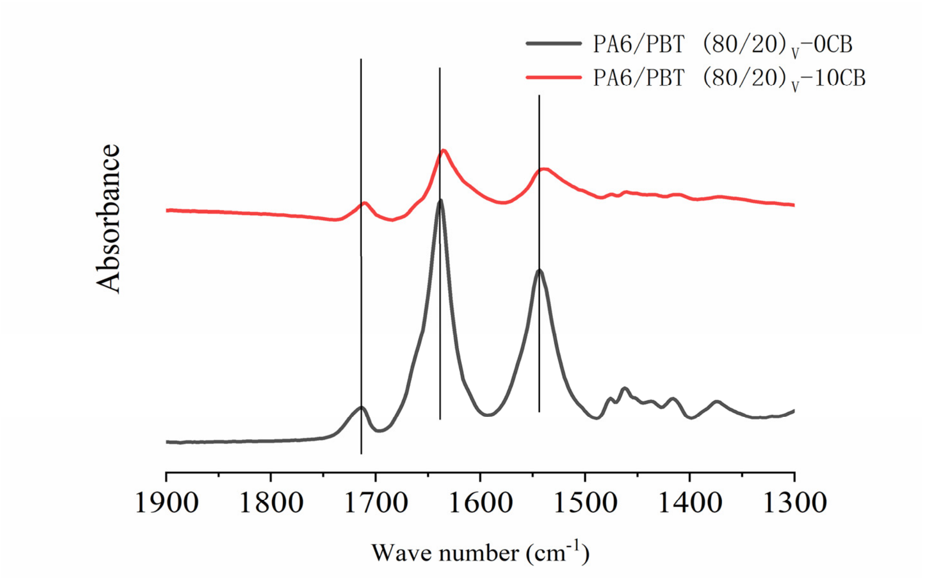

Figure S5. FTIR spectra of PA6/PBT(80/20)-0CB and PA6/PBT(80/20)-10CB composites. The amide I and II bands shifted from 1638.1 to 1635.1 cm $^{-1}$ and from 1543.4 to 1539.1 cm $^{-1}$, and the carbonyl peak shifted from 1713.4 to 1710.1 cm $^{-1}$ after adding 10 vol% CB.

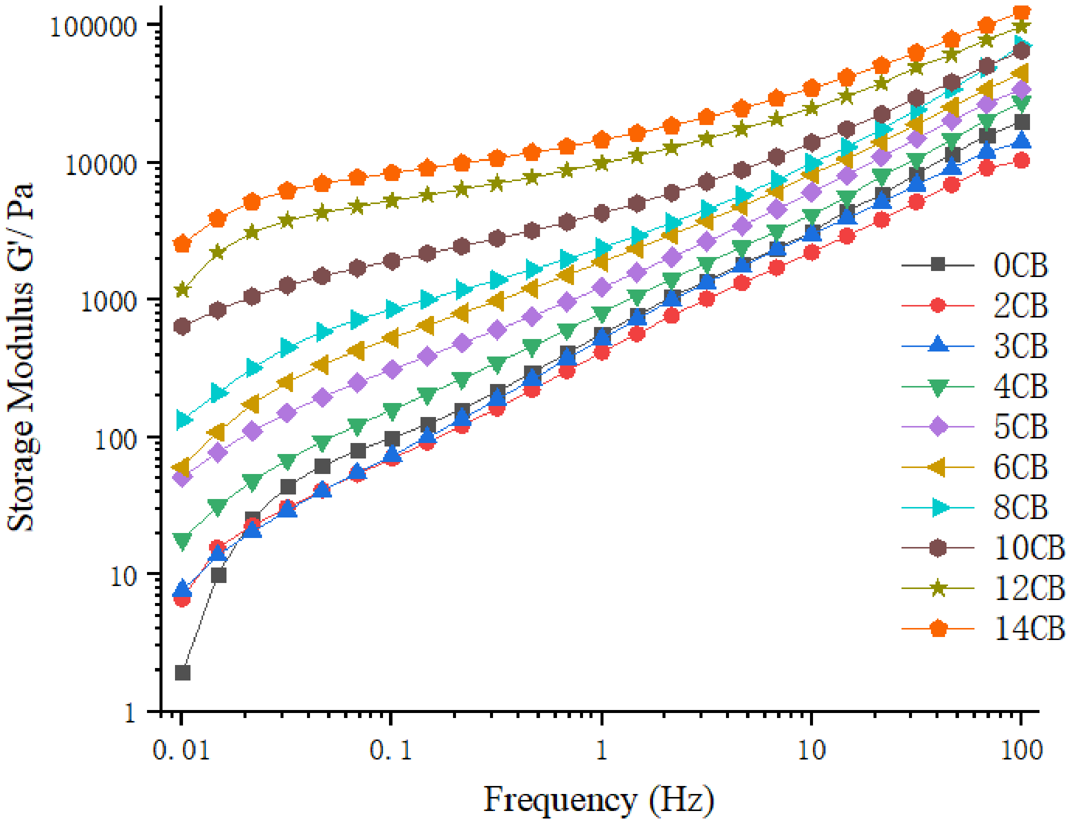

(a) PA6/PBT(80/20)-CB

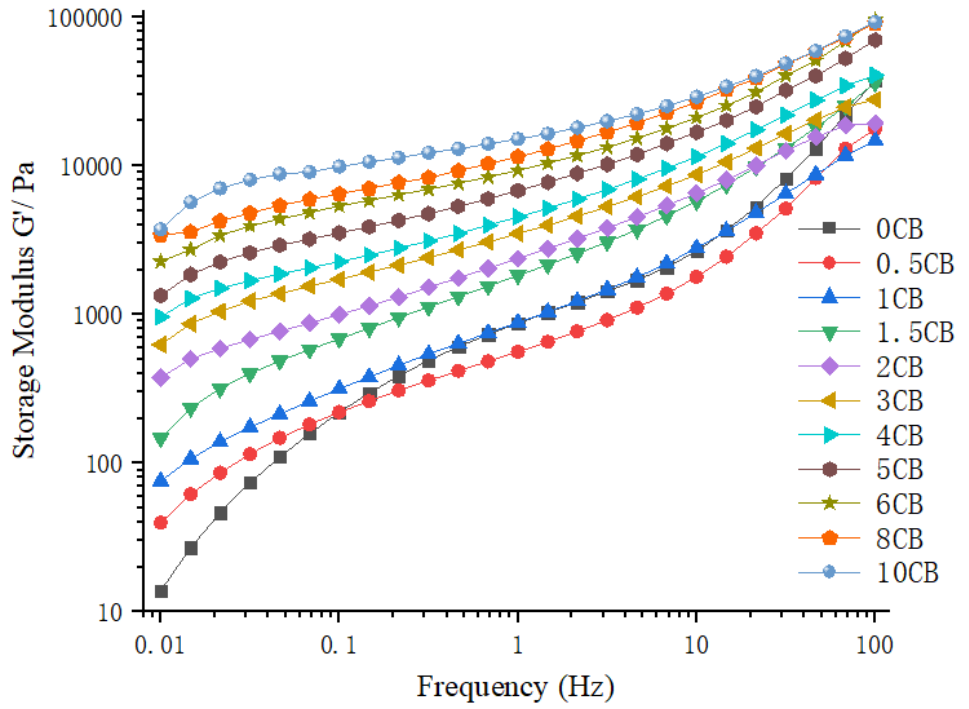

(b) PA6/PBT(50/50)-CB

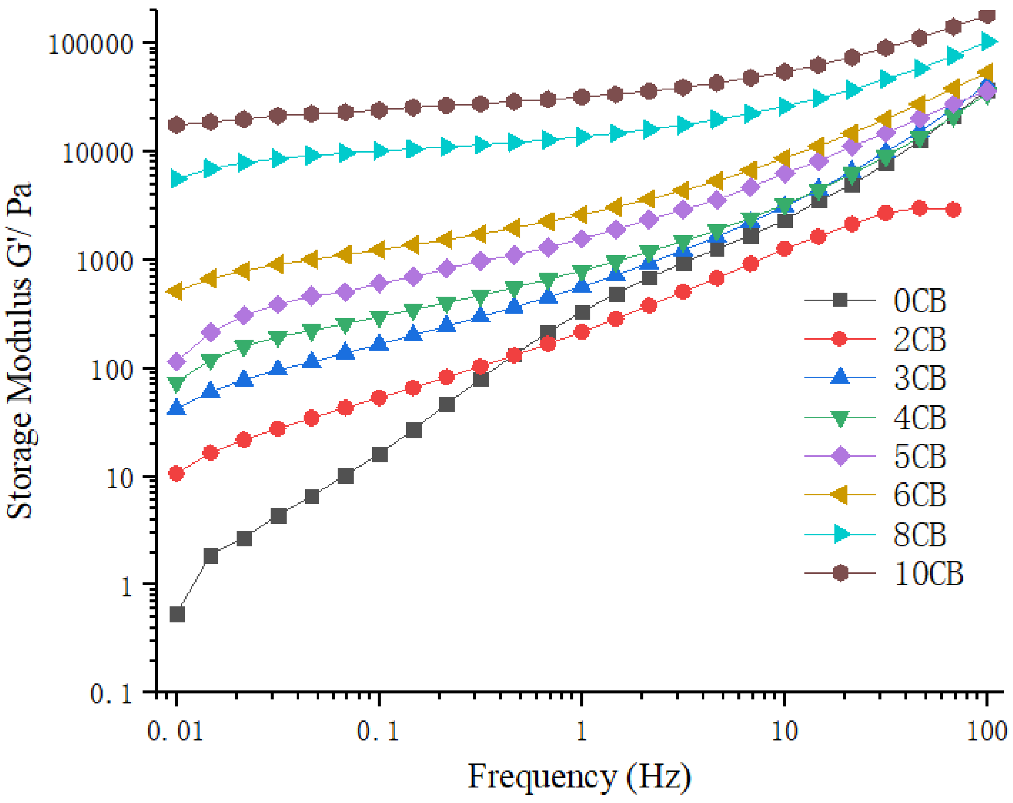

(c) PA6/PBT(20/80)-CB

Figure S6. Storage modulus G' as a function of frequency for PA6/PBT(80/20)-CB (a), PA6/PBT(50/50)-CB (b), and PA6/PBT(20/80)-CB (c) composites with different CB contents.

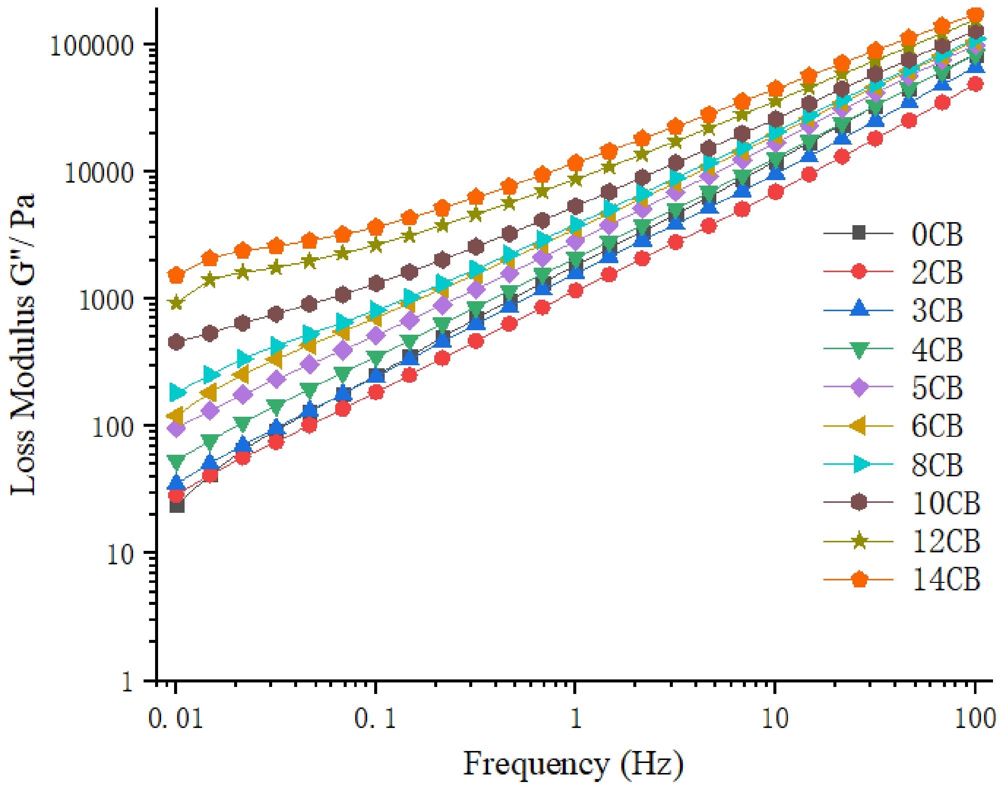

(a) PA6/PBT(80/20)-CB

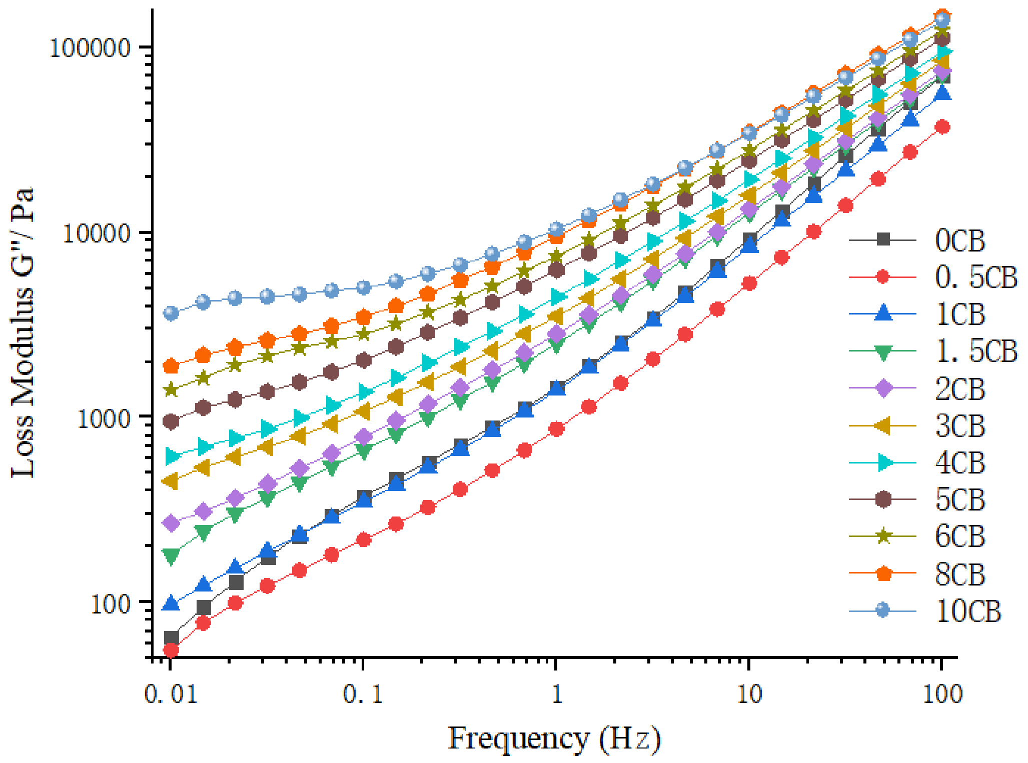

(b) PA6/PBT(50/50)-CB

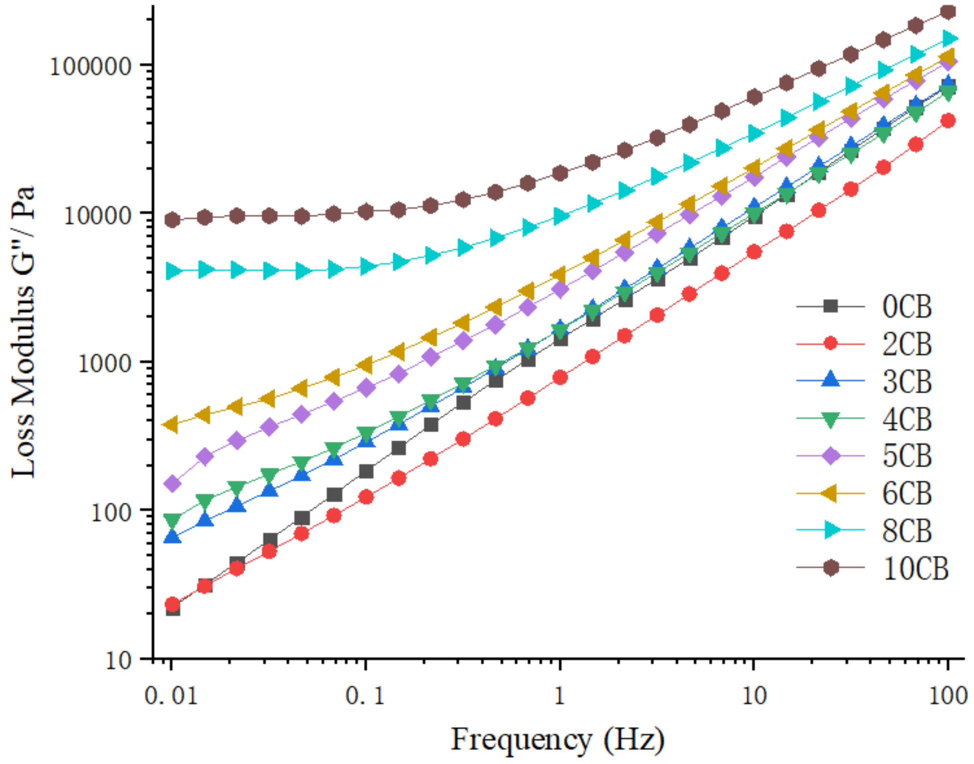

(c) PA6/PBT(20/80)-CB

Figure S7. Loss modulus G'' as a function of frequency for PA6/PBT(80/20)-CB (a), PA6/PBT(50/50)-CB (b), and PA6/PBT(20/80)-CB (c) composites with different CB contents.

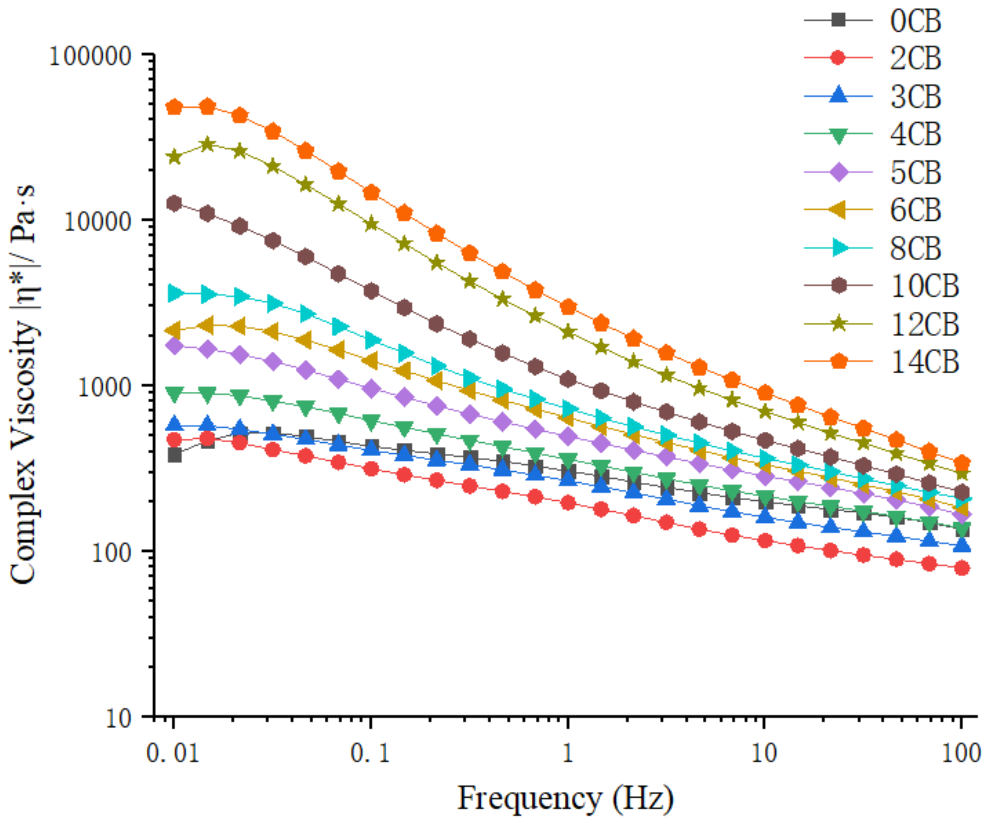

(a) PA6/PBT(80/20)-CB

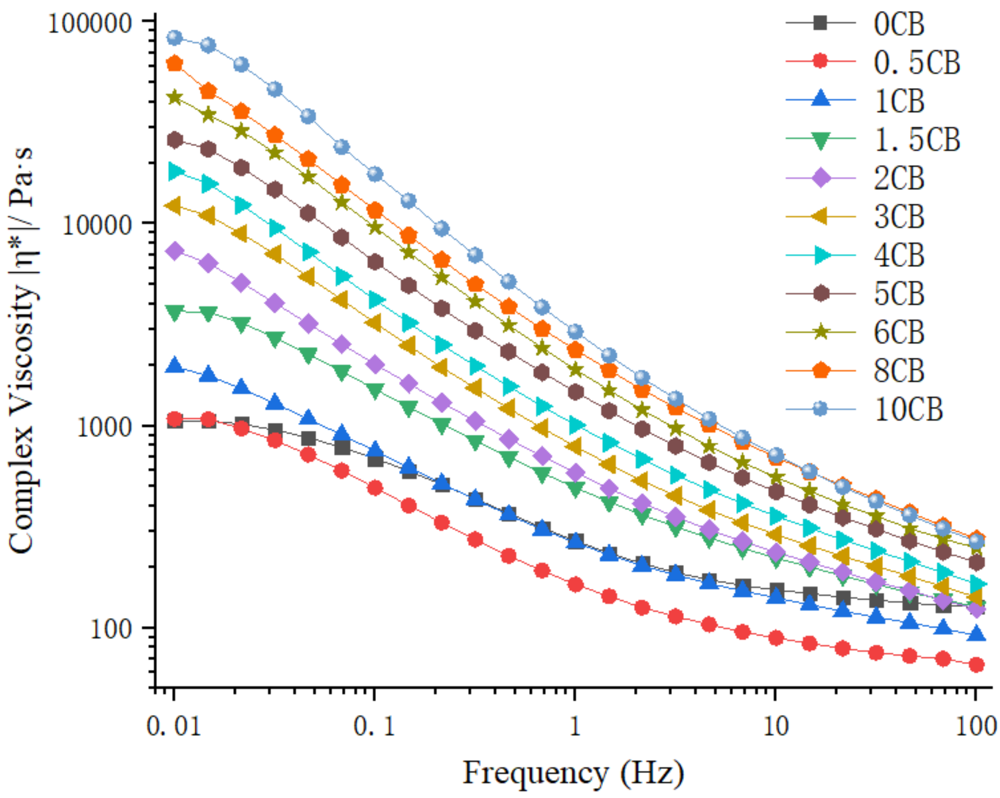

(b) PA6/PBT(50/50)-CB

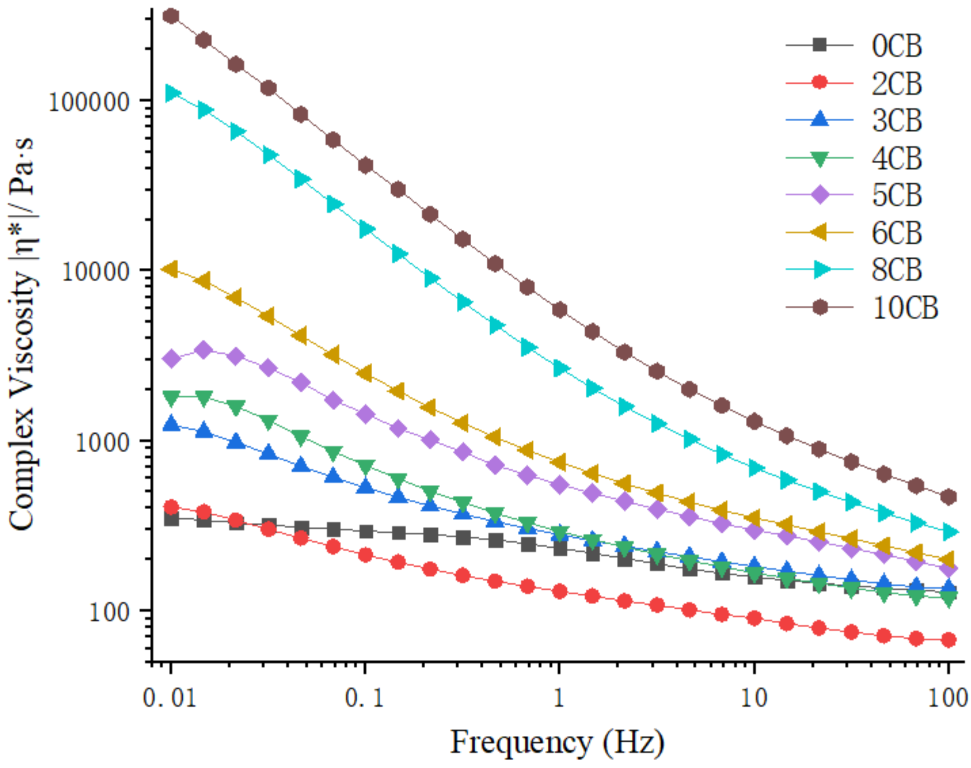

(c) PA6/PBT(20/80)-CB

Figure S8. Complex viscosity |η*| as a function of frequency for PA6/PBT(80/20)-CB

(a), PA6/PBT(50/50)-CB (b), and PA6/PBT(20/80)-CB (c) composites with different CB contents.

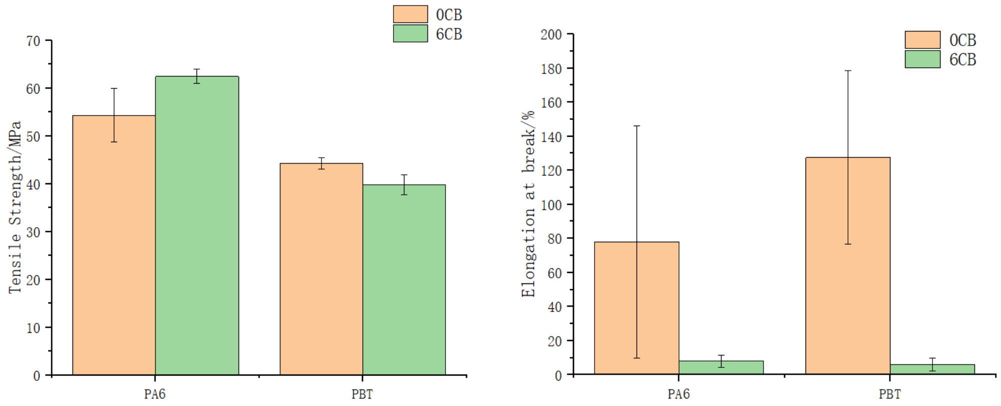

Figure S9. Tensile strengths (a) and elongations at break (b) of PA6 and PBT with CB contents of 0 and 6 vol%.
